# Identifying risk factors for L’Hermitte’s sign after IMRT for head and neck cancer

**DOI:** 10.1186/s13014-018-1015-0

**Published:** 2018-05-04

**Authors:** Hannah M. Laidley, David J. Noble, Gill C. Barnett, Julia R. Forman, Amy M. Bates, Richard J. Benson, Sarah J. Jefferies, Rajesh Jena, Neil G. Burnet

**Affiliations:** 1 Foundation Doctor, Northampton General Hospital, Cliftonville, Northampton, NN1 5BD UK; 2 VoxTox Research Group, Cambridge University Dept. of Oncology, Hutchison/MRC Research Centre, Box 197 Cambridge Biomedical Campus, Cambridge, CB2 0XZ UK; 3 Oncology Centre, Addenbrooke’s Hospital, Cambridge University Hospitals NHS Foundation Trust, Hills Road, Cambridge, CB2 0QQ UK; 4 Cambridge Clinical Trials Unit, Cambridge University Hospitals NHS Foundation Trust, Hills Rd, Cambridge, CB2 0QQ UK; 5 Division of Cancer Sciences, University of Manchester, Manchester Cancer Research Centre, Manchester Academic Health Science Centre, and the Christie NHS Foundation Trust, Manchester, UK

**Keywords:** Chemoradiotherapy, Cisplatin, Head and neck neoplasms, Spinal cord, Transverse myelitis

## Abstract

**Background:**

L’Hermitte’s sign (LS) after chemoradiotherapy for head and neck cancer appears related to higher spinal cord doses. IMRT plans limit spinal cord dose, but the incidence of LS remains high.

**Methods:**

One hundred seventeen patients treated with TomoTherapy™ between 2008 and 2015 prospectively completed a side-effect questionnaire (VoxTox Trial Registration: UK CRN ID 13716). Baseline patient and treatment data were collected. Radiotherapy plans were analysed; mean and maximum spinal cord dose and volumes receiving 10, 20, 30 and 40 Gy were recorded. Dose variation across the cord was examined. These data were included in a logistic regression model.

**Results:**

Forty two patients (35.9%) reported LS symptoms. Concurrent weekly cisplatin did not increase LS risk (*p* = 0.70, OR = 1.23 {95% CI 0.51–2.34}). Of 13 diabetic participants (9 taking metformin), only 1 developed LS (*p* = 0.025, OR = 0.13 {95% CI 0.051–3.27}). A refined binary logistic regression model showed that patients receiving unilateral radiation (*p* = 0.019, OR = 2.06 {95% CI 0.15–0.84}) were more likely to develop LS. Higher V_40Gy_ (*p* = 0.047, OR = 1.06 {95% CI 1.00–1.12}), and younger age (mean age 56.6 vs 59.7, *p* = 0.060, OR = 0.96 {95% CI 0.92–1.00}) were associated with elevated risk of LS, with borderline significance.

**Conclusions:**

In this cohort, concomitant cisplatin did not increase risk, and LS incidence was lower in diabetic patients. Patient age and dose gradients across the spinal cord may be important factors.

**Electronic supplementary material:**

The online version of this article (10.1186/s13014-018-1015-0) contains supplementary material, which is available to authorized users.

## Background

With contemporary techniques, transverse myelitis following radical radiotherapy (RT) for head and neck cancer (HNC) is extremely rare. The QUANTEC paper of 2010 quotes a risk of < 1% at 54Gy with conventional fractionation [1], and recent randomised trials have mandated maximum spinal cord (SC) doses much lower than this [2, 3]. However, milder spinal cord toxicity in the form of L’Hermitte’s sign (LS) may be more prevalent.

LS is characterised by electric-shock sensations down the spine and into limbs on neck movement (particularly flexion). It is a well-recognised symptom of demyelinating conditions such as Multiple Sclerosis (MS) [4], and can also occur as a side effect of radiation to the cervical or thoracic spinal cord [5]. The mechanism is believed to be transient inhibition of oligodendrocyte proliferation leading to reversible demyelination [6, 7]. LS usually develops in the first few months after radiotherapy, and seldom lasts more than 6 months [7–9], but can be unpleasant and distressing for patients. No clear link between radiogenic LS and progressive irreversible myelitis has been established, although delayed radiation-induced myelopathy causing paralysis may be preceded by LS [9].

Historical series, in which patients were treated with conformal, field-based techniques, report a risk of LS following RT for HNC between 3 and 13% [9–11]. More recent work on LS following Intensity Modulated RT (IMRT) for thoracic and head and neck malignancy describes an incidence between 15 and 29% [5, 12, 13]. Since IMRT permits superior sparing of critical organs at risk (OARs), and given our previous understanding of the dose-response relationship of the spinal cord [1, 14], these results are surprising. Recent research has indicated that younger age and higher maximum dose are risk factors, and inferred that concomitant chemotherapy may also be implicated [13, 15].

IMRT can generate steep dose gradients in order to adequately treat target volumes, whilst sparing OARs such as the SC. This often results in inhomogeneous dose distributions across OARs, and work on rat models has suggested that such inhomogeneity may be a risk factor for LS [16]. Whilst some clinical data appears to support this notion [12], no convincing evidence of a ‘bath and shower’ effect has yet been seen [13].

We sought to examine the link between radiation dose, comorbidities, and concomitant systemic therapy and the subsequent development of LS using a logistic regression model, in a cohort of patients with HNC recruited to the VoxTox study.

## Methods

### Study design & patient selection

VoxTox is a longitudinal observational study to collect toxicity data for patients undergoing image-guided IMRT [17]. It received National Research Ethics Service (NRES) Committee East approval in February 2013 (13/EE/0008) and is part of the UK Clinical research network Study Portfolio (UK CRN ID 13716).

Adults undergoing curative RT with daily image guidance (IG) for histologically confirmed HNC are eligible for the broader study. This work included patients with squamous cell carcinomas (SCC’s) and salivary gland tumours (SGT’s) undergoing treatment to the primary site and neck with a minimum of 30 fractions. Prescription doses of 60Gy and above were permitted, and for patients to be included, a *minimum* of ipsilateral nodal levels II and III needed to be included in the neck CTV. As new LS beyond the first year is rare [9], a minimum follow up of 1 year was mandated. The following factors were regarded as exclusion criteria; incomplete baseline screening of past medical history, shorter fractionation schedules (fraction size > 2.17Gy), primary site radiation only, documented neurological comorbidity, non-specific neurological symptoms (e.g. numbness and tingling) at baseline, and insufficient follow up.

### Patient treatment

All patients in this study underwent helical IMRT on the TomoTherapy HiArt™ system with daily IG [18]. Immobilisation was with a thermoplastic shell, and simulation performed on a planning-CT with 3 mm slices. Diagnostic imaging including MRI and PET-CT was rigidly co-registered to aid contour definition. A 3-dose, 30 fraction technique was used for SCCs. Gross primary and nodal disease CTV’s received 65Gy, high-risk elective regions 60Gy and lower risk neck CTVs 54Gy in line with recent trial protocols [2]. Post-operative patients (both SCC’s and SGT’s) underwent adjuvant RT following surgery and received 60Gy in 30 fractions to primary site and elective nodal regions depending on risk. A PTV margin of 5 mm was used for all target volumes. The SC (not canal) was contoured as an OAR, with a 3 mm PRV margin. A maximum dose objective of 46Gy and absolute dose constraint of 50Gy to the SC PRV were defined.

Fit patients (KPS ≥ 80) up to 70 years old with Stage III-IV SCC received concomitant weekly cisplatin (40 mg/m^2^), or cetuximab (400 mg/m_2_ loading dose, 250 mg/m^2^ weekly dose) if specific contraindications to cisplatin existed [19]. Chemotherapy records were retrospectively accessed to define how many cycles of systemic therapy patients received. Baseline characteristics are listed in Table 1.

**Table 1:** Patient characteristics univariate analysis

	Non-LS (*n* = 75)	LS (*n* = 42)	Odds Ratio (95% CI)	*p*-value
Patient characteristics				
Mean age ± SD	59.7 ± 8.7	56.6 ± 11.4	0.96 (0.93–1.01)	0.1^a^
-Difference (95% CI)	3.1 (− 0.9 to 7)			
-Age range	34–79	38–78		
Male	64 (85%)	33 (79%)	0.63 (0.3–2.2)	0.4^b^
-Difference (95% CI)	−6.7% (−21.5 to 8.1)			
Tumour characteristics				
SCC	68 (90.7%)	36 (85.7%)	0.62 (0.3–2.6)	0.2^b^
-Oropharynx	43 (57.3%)	26 (61.9%)		> 0.9
-Oral Cavity	9 (12.0%)	2 (4.8%)		0.4
-Larynx	8 (10.6%)	2 (4.8%)		0.4
-CUP	6 (8.0%)	1 (2.4%)		0.3
-Hypopharynx	1 (1.2%)	2 (4.4%)		> 0.9
-Nasopharynx	1 (1.2%)	3 (6.7%)		> 0.9
Salivary gland	7 (9.3%)	6 (14.3%)	1.6 (0.4–3.9)	> 0.9
Treatment plan				
≥65 Gy prescribed	60 (80.0%)	31 (73.8%)	0.71 (0.4–2.1)	0.7^b^
Unilateral radiotherapy	20 (26.7%)	18 (42.8%)	2.06 (0.6–3.0)	0.07^b^
Cisplatin prescribed	39 (52.0%)	26 (61.9%)	1.5 (0.6–2.6)	0.3^b^
-Cisplatin received	39 (52.0%)	24 (57.1%)	1.23(0.5–2.3)	0.7^b^
Cetuximab prescribed	8 (10.7%)	2 (4.8%)	0.42 (0.1–3.4)	0.3^b^
No chemotherapy	28 (37.3%)	14 (33.3%)	0.84 (0.4–2.1)	0.8^b^
Neurological Risk Factors				
Hypertension	25 (33.3%)	8 (19.0%)	0.47 (0.3–1.79)	0.1^b^
Diabetes	12 (16.0%)	1 (2.4%)	0.13 (0.1–3.3)	0.03^b^

*Abbreviations*: *CI* – confidence interval, *SD* – standard deviation, *CUP* – cancer of unknown primary ^a^Student’s t test, ^b^Fisher’s exact test

### Toxicity assessment

Using both the CTCAE v4.03, and LENT-SOM(A) scales, patients had toxicity assessments undertaken at baseline, and 3, 6 and 12 months post-treatment [20, 21]. Interviews were undertaken by the study research radiographer. Patients were asked about symptoms of LS, and responses were logged digitally at interview into a database within MOSAIQ**®** (Elekta, Stockholm, Sweden) care management software.

CTCAE v4.03 was used for the primary analysis. Symptoms consistent with LS qualified as Grade 1 myelitis. On this basis, patients were categorised as either having LS or not. Previous studies show that the average duration of LS is 4–6 months [10, 11, 13]. We therefore took a single positive response as sufficient to define patients as having developed LS. The LENT-SOM(A) scale was used to grade severity of symptoms from 0 to 4, and this data informed a secondary ordinal analysis. The relevant question from the clinical reporting form is found in supplementary materials (Additional file 1: Figure. S1).

### Spinal cord dosimetry

**Fig. 1:**
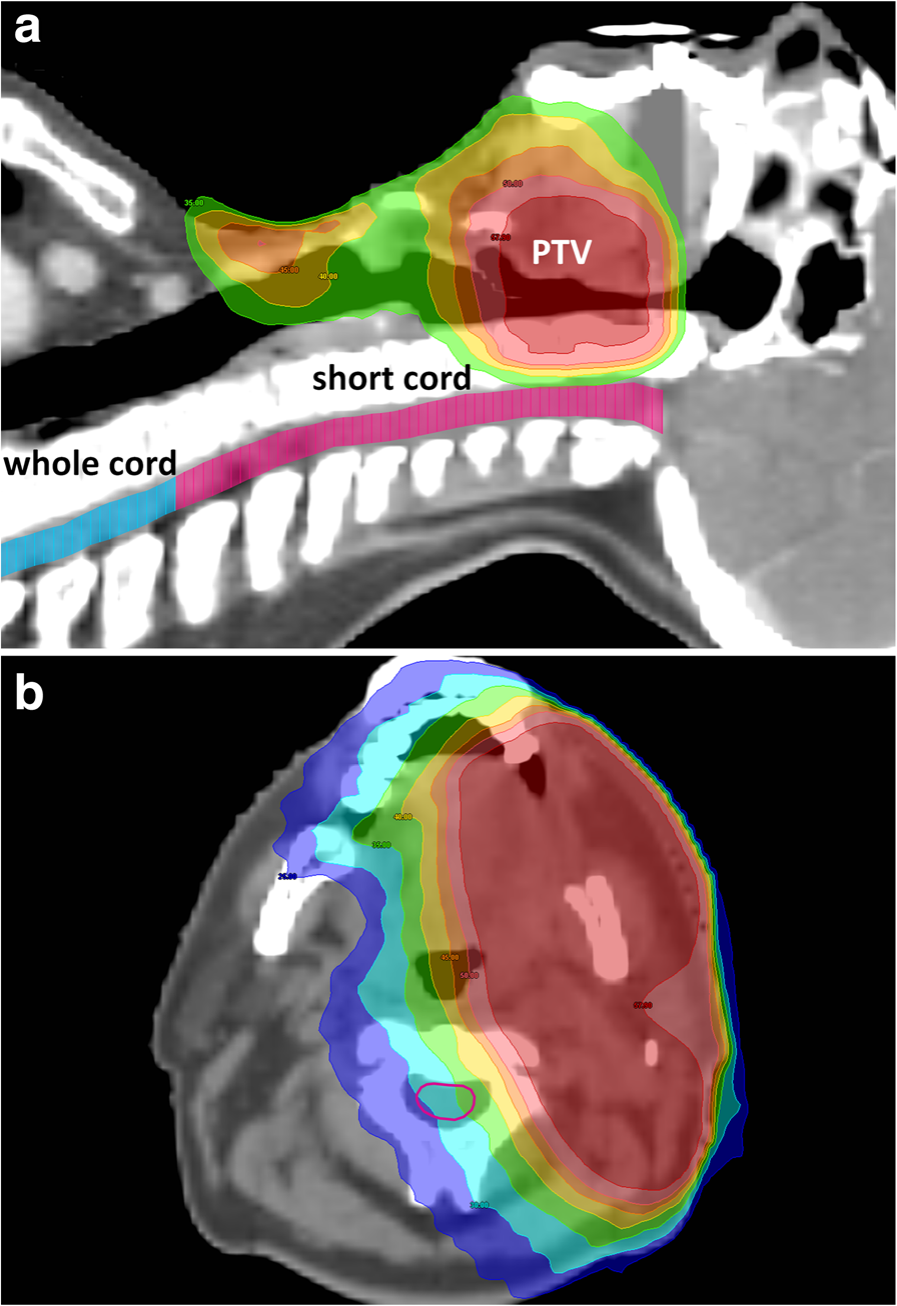
Spinal cord dosimetry **a** ‘whole cord’ shown in blue, ‘short cord’ in pink. **b** axial dose gradient across the cervical cord; max. Left - right gradient 8.3Gy (36.9–28.6Gy). Dose wash; 95% isodose for 60Gy (57.9Gy) dark red, 50Gy light red, 45Gy orange, 40Gy amber, 35Gy green, 30Gy light blue, 25Gy royal blue

Planning CT images, structure sets and dose cubes were reloaded into RT contouring software ProSoma v3.3 (MedCom GmbH, Darmstadt, Germany). To minimise bias from inter-observer contouring variability, the spinal cord was re-contoured for all cases by one observer (DJN). To replicate the methodology of a similar study [13], two separate volumes were created; ‘whole cord’ and ‘short cord’ (Fig. 1a). Whole cord was defined by foramen magnum superiorly, and inferior extent of the planning scan, usually carina, inferiorly. The short cord volume was created by deleting slices caudal to the most inferior PTV slice. ProSoma was used to compute cumulative DVH’s for both structures. The whole cord volume was used to calculate the maximum point dose to the spinal cord (D_max_), and the minimum dose to the 2cm^3^ of the spinal cord receiving the highest dose (D_2cc_), as well as the absolute volumes receiving 10, 20, 30 and 40Gy respectively (V_10Gy_, V_20Gy_, V_30Gy_, V_40Gy_) [13]. The short cord volume was used to calculate mean and median dose to the cervical cord, and relative volumes receiving 10, 20, 30 and 40Gy respectively (V_10%_, V_20%_, V_30%_, V_40%_).

To investigate the effect of dose inhomogeneity [12, 13], DVH data for short cord volumes were used to calculate homogeneity index, defined as (D_2%_ - D_98%_)/D_50%_ [22]. An example patient with LS, and a significant axial dose gradient across the spinal cord, is shown in Fig. 1b.

### Statistics

A refined logistic regression model was developed in three steps: 1) Univariate analyses 2) Logistic regression model 3) Refined logistic regression model. Univariate relationships between LS incidence and baseline parameters, chemotherapy and radiotherapy data were analysed using Fisher’s Exact test if they were binary, student’s t-test if data were parametric (determined by Shapiro-Wilk test), and Mann-Whitney U test if not. Odds ratios with 95% confidence intervals were calculated for independent variables. Estimates of the confidence intervals between medians are shown in Table 2 [23]. A receiver operator characteristic (ROC) curve analysis was used to assess the relationship between number of cycles of cisplatin received, and the incidence of LS.

**Table 2:** Univariate analysis of SC Dose parameters and LS incidence

	Non-LS (*n* = 75)	LS (*n* = 42)	Difference (95% CI)	*p* value (2-tailed)
D_max_	36.4 ± 4.7	36.6 ± 5.3	0.27 (−1.6–2.1)	0.8^a^
D_2cc_	33.1 ± 4.8	33.0 ± 5.6	0.10 (− 1.9–2.1)	0.9^a^
D_mean_	29.4 ± 5.4	28.4 ± 4.9	1.0 (−0.9–2.9)	0.3^a^
HI	0.48 ± 0.27	0.57 ± 0.22	0.093 (0.002–0.2)	0.05^a^
V_10Gy_	14.3 ± 3.3	14.0 ± 2.9	0.35 (− 0.8–1.5)	0.6^a^
V_20Gy_	12.4 ± 3.7	11.5 ± 3.4	0.90 (− 0.4–2.2)	0.3^a^
V_30Gy_	7.6 (1.9–11.3)	4.9 (1.6–8.5)	2.7 (− 0.3–5.7)	0.1^b^
V_40Gy_	0 (0–0.03)	0 (0–0.15)	0 (0–0)	0.9^b^
V_10%_	100 (100–100)	100 (100–100)	0 (0–0)	0.7^b^
V_20%_	99.5 (91.2–100)	96.4 (79.9–99.3)	3.1 (− 1.1–7.4)	0.02^b^
V_30%_	61.60(14.5–86.8)	41.6 (13.9–70.6)	20 (−2–41)	0.1^b^
V_40%_	0 (0–0.19)	0 (0–1.16)	0 (0–0)	0.9^b^

Abbreviations: CI – confidence interval, HI- homogeneity index ^a^Mean ± SD and Student’s t test, ^b^Median, Interquartile range and Mann Whitney U test

Variables were included in a regression model based on three criteria: (i) biological plausibility and evidence from previous studies, (ii) collinearity statistics to reduce confounding, (iii) univariate analysis association at the *p* < 0.15 level. Automated subset selection algorithms were not used, nor was selection based solely on univariate statistics, as these methods may conflate chance effects and reduce the reliability of models in biological systems [24]. Instead variables that fit the three criteria above were used to produce a regression model. Finally, the model was refined by removing all variables where *p* > 0.15 and recalculating the regression model. Statistical analysis was performed using IBM SPSS v23, and R statistical software (R Notebook, R version v3.4.0, R Foundation for Statistical Computing).

## Results

One hundred seventeen patients were included in the final analysis, and 42 patients (35.9%) reported LS symptoms at least once: 29 reported Grade 1 LS, 11 Grade 2, two people had Grade 3 symptoms, and none reported Grade 4. Mean onset of LS symptoms was 5.4 months; median duration was 6 months.

### Univariate analyses

**Fig. 2:**
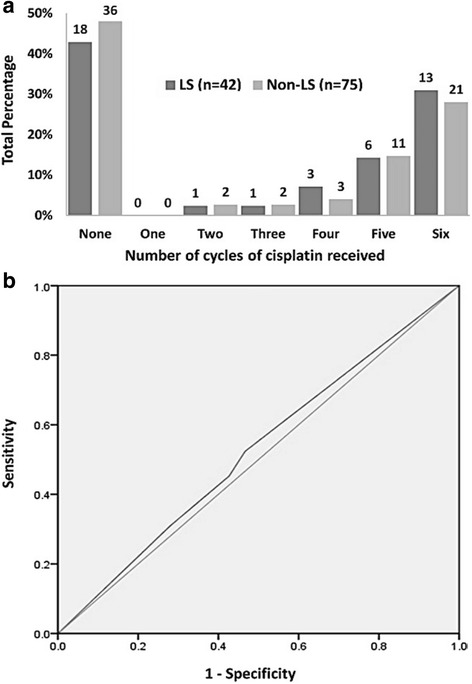
Number of cycles of cisplatin received vs incidence of LS **a** number of cisplatin cycles received by patients with and without LS (absolute numbers above bars). **b** - Receiver Operator Characteristic curve - number of cisplatin cycles received for prediction of LS (AUC = 0.525, 95% CI = 0.416 to 0.634)

Cisplatin prescription (as a binary variable) was not associated with LS development (Table 1). As cisplatin was administered weekly, ROC curve analysis was used to test the hypothesis that patients receiving a greater cumulative dose might be at greater risk, despite the absence of a relationship as a binary variable (Fig. 2). This confirmed that the number of cycles of cisplatin received had no impact on incidence of LS (ROC curve AUC = 0.53, 95% CI 0.43–0.63). However, younger patients were more likely to get cisplatin, (mean age 56.1 +/− 7.8 vs 61.7 +/− 11.1, *p* = 0.0017), whilst diabetic and hypertensive patients were less likely to receive the drug (*p* = 0.0036 and *p* = 0.017 respectively). Univariate analysis showed possible relationships between LS and all 3 factors (Table 1). Therefore, to mitigate the potential for these factors confounding and masking a real effect from concomitant chemotherapy, cisplatin was included in the first iteration of the logistic regression model.

**Fig. 3:**
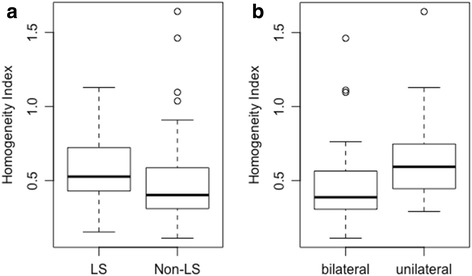
Dose inhomogeneity in patients with and without LS **a** Box and whisker plots showing higher homogeneity index in LS patients **b** higher homogeneity index in patients receiving unilateral neck radiation.

Unilateral neck treatment tends towards significance on univariate analysis, despite mean D_max_ being lower in patients treated unilaterally than those undergoing bilateral neck irradiation (35.4 vs 36.9Gy). This difference did not reach significance at the 5% level (*p* = 0.08, Additional file 1: Figure S2). Homogeneity index was slightly higher in patients with LS (*p* = 0.049) and significantly higher in patients receiving unilateral treatment (*p* = 0.0004) (Table 2, Fig. 3).

Both mean and maximum SC dose was similar in patients with and without LS (Table 2). Only 28.6% of patients who developed LS, but also 26.7% of patients who did not, had SC D_max_ ≥ 40 Gy (*p* = 0.8). Some dose parameters (V_30Gy_, V_40Gy_, V_10%_, V_20%_, V_30%_, V_40%_) were not normally distributed (because they were mostly 0 or 100 – Additional file 1: Figure S3) and could not be transformed to fit a normal distribution. Using the Mann Whitney U test three dose parameters were associated at the *p* < 0.15 level; V_20%_, V_30%_, and V_30Gy_. The latter two were excluded to avoid multi-collinearity (Additional file 1: Figure S4 and Table S1).

Previous studies show that higher SC dose is more likely to cause LS or myelitis [7, 11, 15], with absolute or partial volumes over 40 Gy seemingly most predictive for LS [13]. SC doses in our series are substantially lower than these studies, and the absence of a relationship between V_40Gy_/V_40%_ and LS on univariate analysis (*p* = 0.85 and 0.86 respectively) may have been influenced by the low proportion of patients receiving ≥40 Gy (27%). Those that did had only small volumes of SC receiving 40 Gy (mean 1.44cm^3^). V_40Gy_ metrics were therefore included in the logistic regression model, to directly compare with previous work in the field, to account for possible bias on univariate analysis, and to strengthen our initial assertion if univariate analysis results were reproduced.

### Logistic regressions

A binary logistic regression model (LS vs Non-LS) was produced - Table 3. Age, unilateral vs bilateral radiation (laterality), homogeneity index, diabetes, and hypertension were included as they trended towards significance on univariate analysis (*p* < 0.15, Tables 1 and 2). Cisplatin, V_20%_ and V_40%_ were included as described. The model was refined by removing variables with a *p* value over 0.15. This refined model found 3 predictors of LS with a pre-defined α < 0.05: unilateral neck radiation, higher percentage volumes receiving 40 Gy or more, and absence of diabetes.

**Table 3:** Binary logistic regression with LS vs Non-LS as the dependent variable

Independent Variable	Logistic regression coefficient	Regression *p* value	Odds Ratio^a^ (95% CI)
*Original model*			
Age	−0.034	0.1	0.97 (0.9–1.0)
Laterality	−1.037	0.05	0.35 (0.13–1.0)
Diabetes	2.166	0.06	8.7 (0.9–83)
Hypertension	0.384	0.5	1.5 (0.5–4.3)
Cisplatin	−0.384	0.4	0.68 (0.3–1.8)
V_20%_	−0.007	0.7	0.99 (0.96–1.0)
V_40%_	0.059	0.05	1.06 (1.0–1.1)
Homogeneity Index	1.151	0.3	3.2 (0.3–30)
Constant	−0.129		
*Refined model*			
Age	−0.041	0.06	0.96 (0.9–1.0)
Laterality	−1.048	0.019	0.35 (0.2–0.8)
Diabetes	2.301	0.036	10 (1–85)
V_40%_	0.056	0.047	1.1(1.0–1.1)
Constant	0.200		

*Abbreviations*: *CI* – confidence interval Hosmer and Lemeshow test refined model χ2 (7) = 6.056, *p* = 0.553. Pseudo-R^2^ = 0.14 to 0.18. ^a^Odds ratio per unit increase in variable or for bilateral radiation and being non-diabetic

An ordinal regression (highest grade of LS reported as the dependent variable) was also undertaken to investigate an association with more severe symptoms (Additional file 1: Table S2). This suggested younger age is also a significant predictor of LS (*p* = 0.031).

It is possible that by using relative dose-volume parameters in the primary analysis, small differences in absolute volume were magnified (V_40Gy_ range; 0 to 8.2 cc, V_40%_ range; from 0 to 66.4%). To account for this effect, the analysis was repeated using V_20Gy_ and V_40Gy_ instead of V_20%_ and V_40%_ (Additional file 1: Tables S3 and S4). The outcome was very similar; in this model predictors of LS included younger age (*p* = 0.028), unilateral treatment (*p* = 0.042), higher absolute volumes receiving 40 Gy (*p* = 0.025), and absence of diabetes (*p* = 0.033). V_20Gy_ was predictive in the ordinal regression (*p* = 0.021).

## Discussion

This is the largest prospective study of L’Hermitte’s syndrome in HNC patients in the era of IG-IMRT. LS incidence in our cohort is higher than previously reported (3.6–29%) [5, 11–13, 15], although mean onset and duration of symptoms were similar. The high incidence may be due to its prospective nature, and the fact that a single positive response classified patients as having LS. As LS is transient [9], we believe this definition is justified. Furthermore, these data come from a large, prospectively evaluated cohort of patients treated with a homogeneous protocol including daily IG and positional correction; thus the observed difference in LS incidence is credible.

Pak and colleagues suggest that concurrent neurotoxic chemotherapy may contribute to a higher incidence of LS [13]. The odds ratio for cisplatin on univariate analysis is 1.23 (95% confidence interval is 0.5 to 2.3). Thus, we cannot conclude that there is an effect from cisplatin. It may be that our study was underpowered to detect such an effect, and there may be a confounding effect of separate factors linked with cisplatin use such as age, hypertension and diabetes.

Surprisingly, V_20%_ was significantly lower on univariate analysis in patients with LS, although its effect was insignificant in the multi-variate model. Conversely, V_40Gy_ and V_40%_ were insignificant on univariate analysis but significant according to the logistic regression model, consistent with previous work [13]. Of note, TomoTherapy plans confer excellent spinal cord sparing, meaning only 27.3% of all the patients in our cohort had a partial cord volume receiving 40 Gy or more. Therefore, our V_40Gy_ results should be interpreted with caution. Mean D_max_ in our cohort was 36.4 Gy compared to 39.1 to 42.5 Gy in similar studies using VMAT and IMRT respectively [12, 13], yet more patients in our cohort reported LS than in these studies. Interestingly, a study on 105 patients receiving thoracic IMRT for lymphoma reported a mean D_max_ of 33.5 Gy and had an LS incidence of 29% [5], also suggesting factors other than dose may be important. According to our multi-variate analysis, age, diabetes, and unilateral neck radiation may be related factors, although given the sample size and degrees of freedom in the model, *p*-values for all factors should be considered borderline significant, and interpreted with caution.

Patients developing LS were younger than patients without LS. The difference was insignificant on univariate analysis and binary logistic regression, but significant in the ordinal regression, suggesting younger patients have more severe symptoms if they do develop LS. These findings are not new: Mul and co-workers found a mean age of 52 in LS patients compared with 61 in non-LS patients [15], whilst Leung et al. found a decreased risk in those over 60 [10]. Although younger patients were more likely to receive cisplatin (*p* = 0.0017) they were not more likely to receive a higher maximum or mean SC dose (*r* = 0.030 and − 0.203 respectively, Pearson correlation coefficient).

Intriguingly, our data suggest that patients with diabetes are less likely to develop LS, a previously unreported finding. It should be noted that 10.2% of our cohort had diabetes compared to 3.9% and 4.1% in similar studies [13, 15]. Nine of 13 diabetic patients in our cohort took metformin (the one diabetic patient with LS also took metformin). This drug has been suggested to have anti-inflammatory and anti-oxidant neuroprotective effects in mouse models of MS [25, 26], whilst a significant anti-inflammatory effect of metformin and pioglitazone has also been shown in patients with MS and metabolic syndrome [27]. However, more investigation would be needed to ascertain whether these benefits are also seen in radiation-induced demyelination.

Lastly, patients with LS were significantly more likely to have had unilateral neck radiation. A ‘bath and shower’ effect, whereby radiation tolerance is reduced if an area of high dose is surrounded by an area of low dose, was first demonstrated in rat spinal cords [16, 28]. It is hypothesised that low dose radiation prevents oligodendrocyte migration to repair damage, and can alter gene expression [29, 30]. This effect was sought, but not found in a previous clinical study [13]. However, Ko and colleagues observed LS exclusively in patients that received unilateral radiotherapy (5 of 33 patients), and suggest that axial dose inhomogeneity may contribute to the development of LS [12]. Other authors postulate that anterior-posterior dose gradients may be significant because of spinothalamic tract damage [31].

In addition to a relationship between unilateral neck irradiation and LS, we also found that unilateral neck treatment plans had much more inhomogeneous SC dosimetry. Although this inhomogeneity was not an independent risk factor for LS in the multi-variate model, this may be due to the close association with treatment laterality, and the possibility of diluted statistical power within the model. Our interpretation of these data are as follows; firstly, to corroborate previous findings of higher LS risk in patients undergoing unilateral neck treatment, secondly to suggest that inhomogeneous SC dose distributions may be a mechanistic factor in this effect, and finally that a paradoxically rising incidence of LS may in part be due to the greater SC dose inhomogeneity that IMRT confers. It is clear however that understanding of neurological response to complex dose distributions is incomplete.

## Conclusion

The incidence of LS in this study remains paradoxically higher than previously reported, despite modern IMRT techniques delivering low SC doses. We found no increased risk from concomitant cisplatin, but confirmed previously reported higher risk in younger patients, and with higher volumes of SC receiving ≥40 Gy. Diabetes appeared to reduce risk, and unilateral neck treatment was associated with LS. Greater SC dose inhomogeneity may explain this finding, but further work on neurological response to complex dose distributions is required.

## Additional file

Additional File 1: **Figure S1.** The relevant question from the clinical reporting form Shows the question patients were asked to grade severity of LS from 1 to 4. **Figure S2.** Maximum spinal cord dose in patients with unilateral and bilateral neck radiation Box and whisker plot showing no difference in D_max_ for patients with unilateral and bilateral neck radiation. **Figure S3.** Dose parameters in patients with no LS symptoms (unshaded, *n* = 75), and with LS (shaded, *n* = 42) **A** – Dose to spinal cord. **B** – Volume of spinal cord receiving 10, 20, 30, and 40 Gy. **C** – Percentage of spinal cord receiving 10, 20, 30, and 40 Gy. **Figure S4.** Dose parameter multi-collinearity plots. **A** – V_20%_ vs V_30%_. **B** – V_20%_ vs V_40%_ Scatter plots showing significant multicollinearity between V_20%_ and V_30%_, but less collinearity between V_20%_ and V_40%_. **Table S1.** Collinearity statistics for models containing V_20%_, V_30%_, and V_40%_ Tables showing variance inflation factor and tolerance statistics for logistic regression models containing A V_20%_, V_30%_, and V_40%_ (high collinearity); and B V_20%_ and V_40%_ (low collinearity). **Table S2.** Ordinal logistic regression with highest reported grade of LS as the dependent variable Logistic regression output showing younger age and absence of diabetes are significantly associated with higher grade LS. **Table S3.** Binary logistic regression with LS vs Non-LS as the dependent variable, and absolute dose volumes (in cc). Logistic regression output showing that using absolute volume or percentage volume makes little difference to the predictive power of the model or the odds ratio for variables in the refined model. **Table S4.** Ordinal logistic regression with highest reported grade of LS as the dependent variable, and absolute dose volumes (in cc). Logistic regression output showing that using absolute volume or percentage volume makes little difference to the predictive power of the model or the odds ratio for variables in the refined model. (PDF 234 kb)

## Abbreviations

CI: Confidence interval
CT: Computed tomography
CTCAE: Common terminology criteria for adverse events
CTV: Clinical target volume
D_2cm_^3^: The minimum dose to the 2cm^3^ of the spinal cord receiving the highest dose
D_max_: Maximum point dose to the spinal cord
EORTCQLQ: European organisation for research and treatment of cancer quality of life questionnaire
HNC: Head and neck cancer
IBM SPSS: International business machines corporation statistical package for social sciences
IG: Image guidance
IMRT: Intensity modulated radiotherapy
KPS: Karnofsky performance status
LENTSOM(A): late effects normal tissue task force – subjective, objective, management, analytic
LS: L’Hermitte’s sign
MRI: Magnetic resonance imaging
MS: Multiple sclerosis
OAR: Organ at risk
OR: Odds ratio
PET: Positron emission tomography
PRV: Planning organ at risk volume
PTV: Planning target volume
ROC: Receiver operator characteristic
RT: Radiotherapy
SC: Spinal cord
SCC: Squamous cell carcinoma
SGT: Salivary gland tumour
V_40%_: Percentage volume of spinal cord receiving 40Gy or more
V_40Gy_: Absolute volume of spinal cord receiving 40Gy or more
VMAT: Volumetric modulated arc therapy
