# Supplementary material for: Identifying risk factors for L’Hermitte’s sign after IMRT for head and neck cancer
Source: Radiat Oncol. 2018 May 4;13:84. doi: 10.1186/s13014-018-1015-0 (PMC5936022; doi:10.1186/s13014-018-1015-0)
Supplement: Supplementary file 1 — Figure S1. The relevant question from the clinical reporting form Shows the question patients were asked to grade severity of LS from 1 to 4. Figure S2. Maximum spinal cord dose in patients with unilateral and bilateral neck radiation Box and whisker plot showing no difference in Dmax for patients with unilateral and bilateral neck radiation. Figure S3. Dose parameters in patients with no LS symptoms (unshaded, n = 75), and with LS (shaded, n = 42) A – Dose to spinal cord. B – Volume of spinal cord receiving 10, 20, 30, and 40 Gy. C – Percentage of spinal cord receiving 10, 20, 30, and 40 Gy. Figure S4. Dose parameter multi-collinearity plots. A – V20% vs V30%. B – V20% vs V40% Scatter plots showing significant multicollinearity between V20% and V30%, but less collinearity between V20% and V40%. Table S1. Collinearity statistics for models containing V20%, V30%, and V40% Tables showing variance inflation factor and tolerance statistics for logistic regression models containing A V20%, V30%, and V40% (high collinearity); and B V20% and V40% (low collinearity). Table S2. Ordinal logistic regression with highest reported grade of LS as the dependent variable Logistic regression output showing younger age and absence of diabetes are significantly associated with higher grade LS. Table S3. Binary logistic regression with LS vs Non-LS as the dependent variable, and absolute dose volumes (in cc). Logistic regression output showing that using absolute volume or percentage volume makes little difference to the predictive power of the model or the odds ratio for variables in the refined model. Table S4. Ordinal logistic regression with highest reported grade of LS as the dependent variable, and absolute dose volumes (in cc). Logistic regression output showing that using absolute volume or percentage volume makes little difference to the predictive power of the model or the odds ratio for variables in the refined model. (PDF 234 kb) [file 13014_2018_1015_MOESM1_ESM.pdf]

Supplementary Material

Figure S1: *The relevant question from the clinical reporting form*

Do you experience tingling or electric shock sensation down your back?

0. Never
1. Occasional and mild
2. Sometimes but bearable
3. Often present or severe
4. Constant and very severe

Figure S2: *Maximum spinal cord dose in patients with unilateral and bilateral neck radiation*

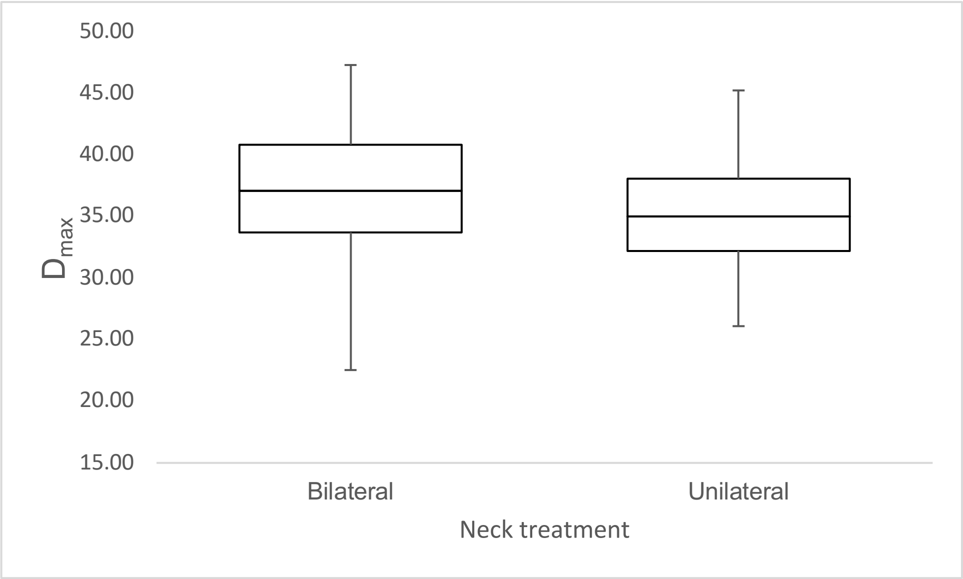

Figure S3: *Dose parameters in patients with no LS symptoms (unshaded, n=75), and with LS (shaded, n=42)* ***A*** *– Dose to spinal cord.* ***B*** *– Volume of spinal cord receiving 10, 20, 30, and 40 Gy.* ***C*** *– Percentage of spinal cord receiving 10, 20, 30, and 40 Gy*

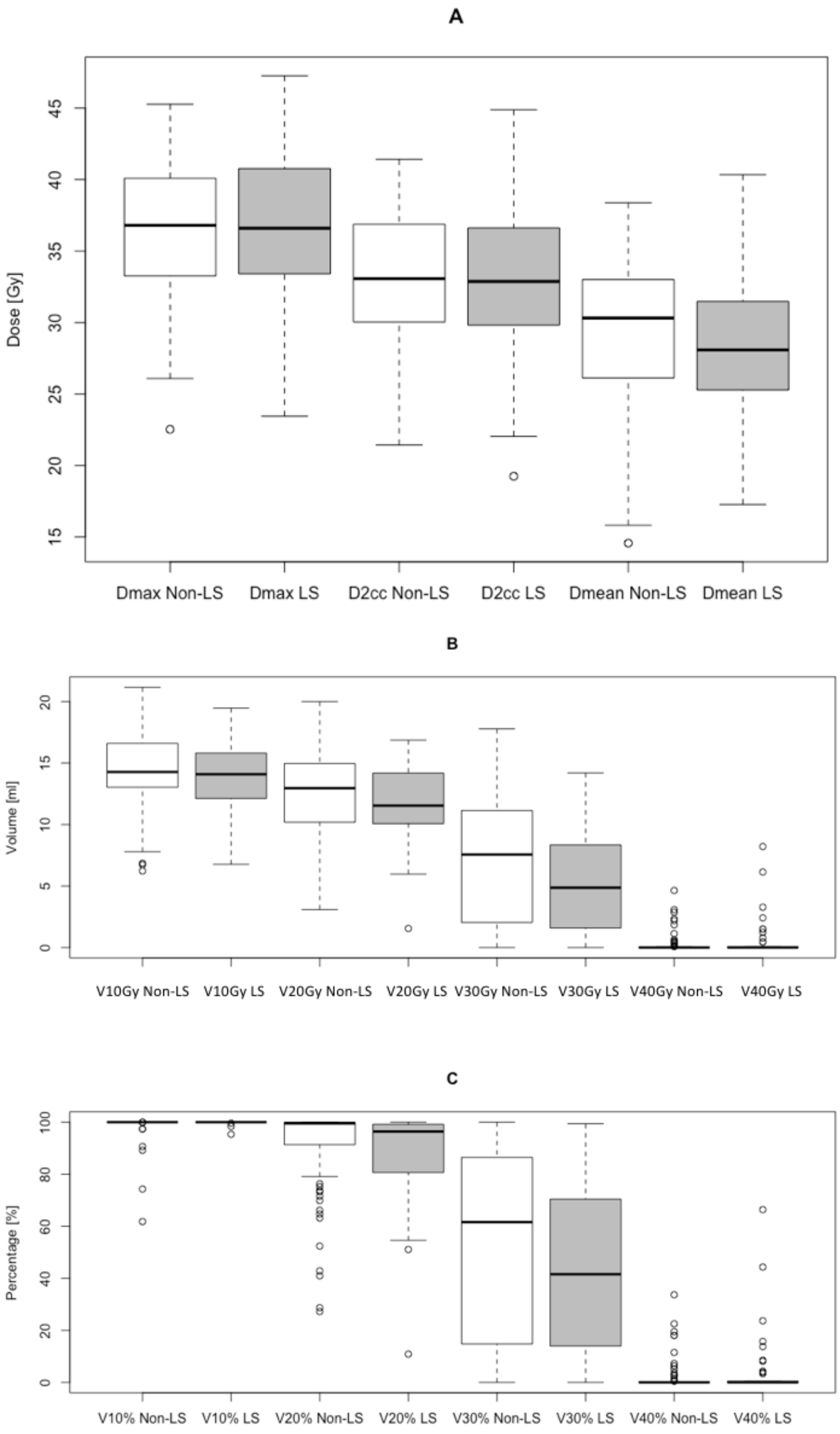
A
Dose [Gy]
15
20
25
30
35
40
45
Dmax Non-LS
Dmax LS
D2cc Non-LS
D2cc LS
Dmean Non-LS
Dmean LS
B
Volume [ml]
0
5
10
15
20
V10Gy Non-LS
V10Gy LS
V20Gy Non-LS
V20Gy LS
V30Gy Non-LS
V30Gy LS
V40Gy Non-LS
V40Gy LS
C
Percentage [%]
0
20
40
60
80
100
V10% Non-LS
V10% LS
V20% Non-LS
V20% LS
V30% Non-LS
V30% LS
V40% Non-LS
V40% LS

Figure S4: *Dose parameter multi-collinearity scatter plots.* ***A*** *– $V_{20\%}$ vs $V_{30\%}$.* ***B*** *– $V_{20\%}$ vs $V_{40\%}$*

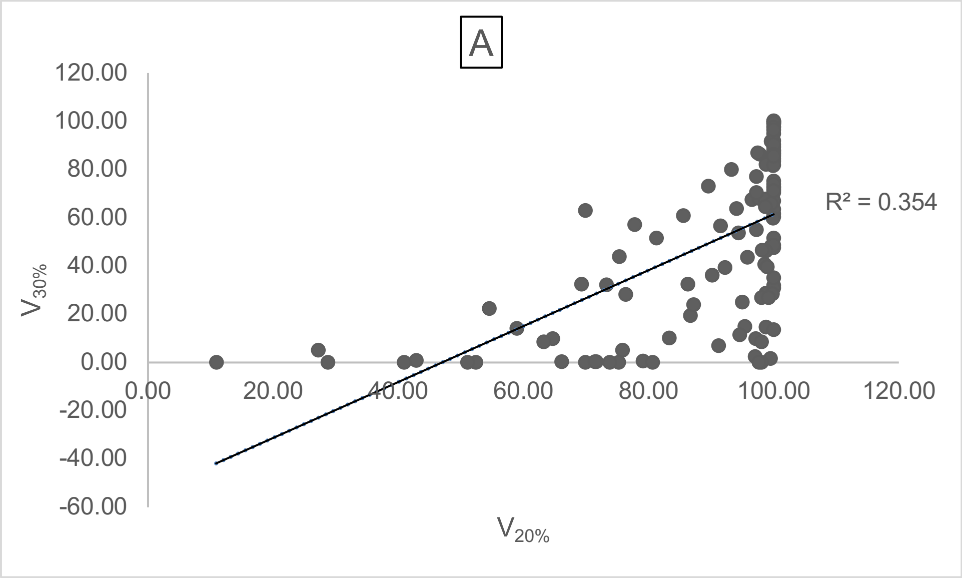

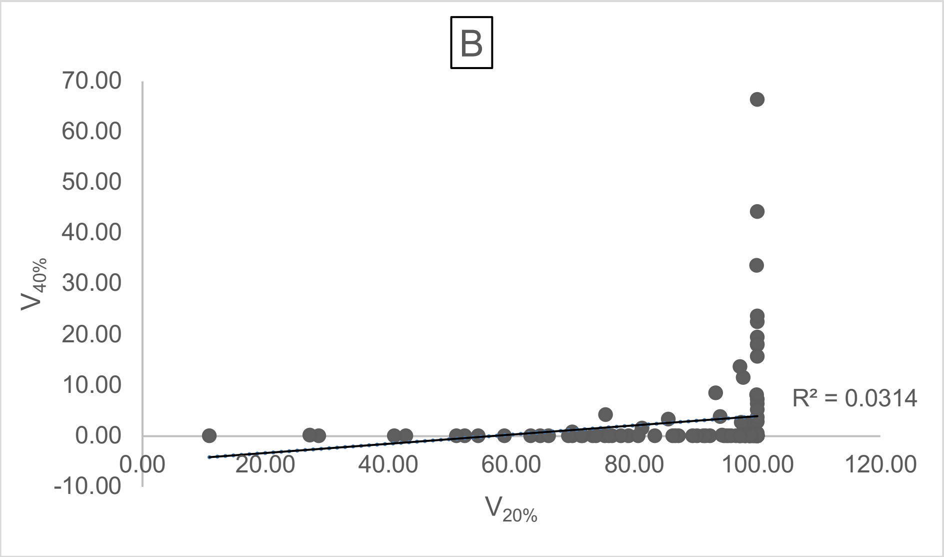

Table S1 (A&B): *Collinearity Statistics for models containing V20%, V30%, and V40%*

**A** Model	Unstandardized Coefficients		Standardized Coefficients	t	Sig.	Collinearity Statistics	
	B	Std. Error	Beta			Tolerance	VIF
(Constant)	.434	.244		1.782	.077		
$V_{20\%}$	.001	.003	.021	.188	.851	.642	1.556
$V_{30\%}$	-.003	.002	-.235	-1.929	.056	.562	1.778
$V_{40\%}$	.012	.005	.220	2.213	.029	.843	1.186

a. Dependent Variable: LS

**B** Model	Unstandardized Coefficients		Standardized Coefficients	t	Sig.	Collinearity Statistics	
	B	Std. Error	Beta			Tolerance	VIF
(Constant)	.594	.232		2.565	.012		
$V_{20\%}$	-.003	.003	-.106	-1.130	.261	.969	1.032
$V_{40\%}$	.008	.005	.151	1.609	.110	.969	1.032

a. Dependent Variable: LS

Table S2: *Ordinal logistic regression with highest reported grade of LS as the dependent variable.*

**Independent Variable**	**Logistic regression coefficient**	**Regression p value**	**Odds Ratio[a] (95% CI)**
	Original model		
**Age**	-0.049	0.027	0.952 (0.91-0.99)
**$V_{20\%}$**	-0.009	0.559	0.99 (0.96-1.23)
**$V_{40\%}$**	0.032	0.157	1.032 (0.89-1.08)
**HI**	0.903	0.399	2.5 (0.30 – 20)
**Laterality**	-0.572	0.230	0.564 (0.22-1.44)
**Diabetes**	1.738	0.090	5.686 (0.76-42.52)
**Hypertension**	0.482	0.355	1.619 (0.58-4.50)
**Cisplatin**	0.051	0.912	1.052 (0.43-2.58)
	Refined model		
**Age**	-0.044	0.031	0.957 (0.92-1.00)
**Diabetes**	1.875	0.065	6.521 (0.89-47.61)

Chi squared test $\chi 2$ (2) =10.22, p = 0.006. Pseudo-R^2 = 0.047 to 0.099.

[a]*Odds ratio per unit increase in variable or for bilateral radiation and being non-diabetic.*

Table S3: *Binary logistic regression with LS vs Non-LS as the dependent variable, and absolute dose volumes (in cc).*

**Independent Variable**	**Logistic regression coefficient**	**Regression p value**	**Odds Ratio[a] (95% CI)**
		Original model	
**Age**	-0.042	0.080	0.959 (0.92-1.01)
**Laterality**	-1.096	0.039	0.334 (0.12-0.95)
**Diabetes**	2.194	0.063	8.972 (0.89-90.52)
**Hypertension**	0.439	0.429	1.551 (0.52-4.60)
**Cisplatin**	-0.495	0.335	0.609 (0.22-1.67)
**V_{20Gy}**	-0.114	0.094	0.893 (0.78-1.02)
**V_{40Gy}**	0.532	0.025	1.702 (1.07-2.71)
**HI**	0.992	0.276	2.70 (0.45-16.10)
**Constant**	1.093		
		Refined model	
**Age**	-0.051	0.028	0.951 (0.91-1.00)
**Laterality**	-0.966	0.042	0.381 (0.15-0.96)
**Diabetes**	2.388	0.033	10.9 (1.21-98.5
**V_{20Gy}**	-0.119	0.056	0.89 (0.79-1.0)
**V_{40Gy}**	0.513	0.025	1.67 (1.07-2.62)
**Constant**	2.053		

Hosmer and Lemeshow test $\chi2$ (8) = 6.770, p = 0.562. Pseudo-R^2 = 0.16 to 0.22.

[a]Odds ratio per unit increase in variable or for bilateral radiation and being non-diabetic.

Table S4: *Ordinal logistic regression with highest reported grade of LS as the dependent variable, and absolute dose volumes (in cc).*

**Independent Variable**	**Logistic regression coefficient**	**Regression p value**	**Odds Ratio[a] (95% CI)**
	Original model		
**Age**	-0.057	0.011	0.945 (0.90-0.99)
**V_{20Gy}**	-0.123	0.051	0.884 (0.78-1.00)
**V_{40Gy}**	0.299	0.084	1.349 (0.96-1.89)
**HI**	0.762	0.363	2.143 (0.41-11.07)
**Laterality**	-0.610	0.200	0.543 (0.21-1.38)
**Diabetes**	1.622	0.119	5.063 (0.66-38.78)
**Hypertension**	0.568	0.283	1.765 (0.63-4.98)
**Cisplatin**	-0.054	0.907	0.947 (0.38-2.36)
	Refined model		
**Age**	-0.062	0.005	0.940 (0.90-0.98)
**Diabetes**	1.790	0.074	5.989 (0.84-42.69)
**V_{20Gy}**	-0.132	0.021	0.876 (0.78-0.98)
**V_{40Gy}**	0.288	0.079	1.334 (0.97-1.84)

Chi squared test $\chi 2$ (4) =17.31, p = 0.002. Pseudo-R^2 = 0.08 to 0.16.

[a]Odds ratio per unit increase in variable or for bilateral radiation and being non-diabetic.
